# Effects of preoperative bicarbonate and lactate levels on short-term outcomes and prognosis in elderly patients with colorectal cancer

**DOI:** 10.1186/s12893-023-02039-x

**Published:** 2023-05-15

**Authors:** Xiao-Yu Liu, Zi-Wei Li, Bin Zhang, Fei Liu, Wei Zhang, Dong Peng

**Affiliations:** Department of Gastrointestinal Surgery, the First Affiliated Hospital of Chongqing Medical University, Chongqing, 400016 China

**Keywords:** Bicarbonate, Lactate levels, Prognosis, Elderly patients, Colorectal cancer

## Abstract

**Purpose:**

The aim of this study was to analyze the effect of preoperative bicarbonate and lactate levels (LL) on the short-term outcomes and prognosis in elderly (≥ 65 years) patients with colorectal cancer (CRC).

**Methods:**

We collected the information of CRC patients from Jan 2011 to Jan 2020 in a single clinical center. According to the results of preoperative blood gas analysis, we divided patients into the higher/lower bicarbonate group and the higher/lower lactate group, and compared their baseline information, surgery-related information, overall survival (OS) and disease-free survival (DFS).

**Results:**

A total of 1473 patients were included in this study. Comparing the clinical data of the higher/lower bicarbonate group and the higher/lower lactate group, the lower group were older (p < 0.01), had higher rates of coronary heart disease (CHD) (p = 0.025), a higher proportion of colon tumors (p < 0.01), larger tumor size (p < 0.01), higher rates of open surgery (p < 0.01), more intraoperative blood loss (p < 0.01), higher overall complications (p < 0.01) and 30-day deaths (p < 0.01). The higher LL patients had more male patients (p < 0.01), higher body mass index (BMI) (p < 0.01) and drinking rates (p = 0.049), higher rates of type 2 diabetes mellitus (T2DM) (p < 0.01) and lower rates of open surgery (p < 0.01). In multivariate analysis, age (p < 0.01), BMI (p = 0.036), T2DM (p = 0.023), and surgical methods (p < 0.01) were independent risk factors of overall complications. The independent risk factors for OS included age (p < 0.01), tumor site (p = 0.014), tumor stage (p < 0.01), tumor size (p = 0.036), LL (p < 0.01), and overall complications (p < 0.01). The independent risk factors of DFS included age (p = 0.012), tumor site (p = 0.019), tumor stage (p < 0.01), LL (p < 0.01), and overall complications (p < 0.01).

**Conclusion:**

Preoperative LL significantly affected postoperative OS and DFS of CRC patients, but bicarbonate might not affect the prognosis of CRC patients. Therefore, surgeons should actively focus on and adjust the LL of patients before surgery.

**Supplementary Information:**

The online version contains supplementary material available at 10.1186/s12893-023-02039-x.

## Introduction

According to Global Cancer Statistics 2020, the global incidence of colorectal cancer (CRC) was increased year by year, and it has now become the second leading cause of cancer-related death after lung cancer and the third leading cause of mortality worldwide. [1–3] The incidence of CRC is 38.7 per 100,000 and the mortality rate is 13.9 per 100,000 [4]. The treatment methods for CRC including surgery, radiotherapy, chemotherapy, immunotherapy, and targeted therapy [5, 6]. Radical resection was still the standard treatment for CRC [7, 8]. Elderly patients usually had poor physical function and more comorbidities [9]. Despite tremendous advances in surgical techniques and perioperative management, postoperative morbidity and mortality of elderly patients remained significantly higher after major abdominal surgery [10].

The number of people reaching old age increased rapidly globally according to the United Nations World Population Prospects, people aged 65 and over now accounted for more than 20% of the world’s population [9]. As the average survival age increased, the number of elderly CRC patients undergoing surgery would also continue to increase. Studies have shown that age was an independent risk factor for the occurrence of CRC and for postoperative complications and mortality. [11–14].

The study of postoperative short-term outcomes and prognostic risk factors in elderly CRC patients has been a hot topic. Studies have reported that preoperative CA19-9 level, ASA grade, low prognostic nutritional index and malnutrition were related to the prognosis of elderly CRC patients. [15–17] Bicarbonate and lactate levels (LL) were important components of blood gas analysis. However, there was no clear study on whether preoperative bicarbonate and LL affected the prognosis of elderly CRC patients. Therefore, the aim of this study was to analyze the effect of preoperative bicarbonate and LL on short-term outcomes and prognosis in elderly (≥ 65 years) patients with CRC.

## Methods

### Patients

We collected the information of CRC patients from Jan 2011 to Jan 2020 in a single clinical center. The study was approved by the ethics committee of our institution (The First Affiliated Hospital of Chongqing Medical University, 2022-K205), and all patients signed informed consent forms. This study was conducted in accordance with the World Medical Association Declaration of Helsinki as well.

### Inclusion and exclusion criteria

**Fig. 1:**
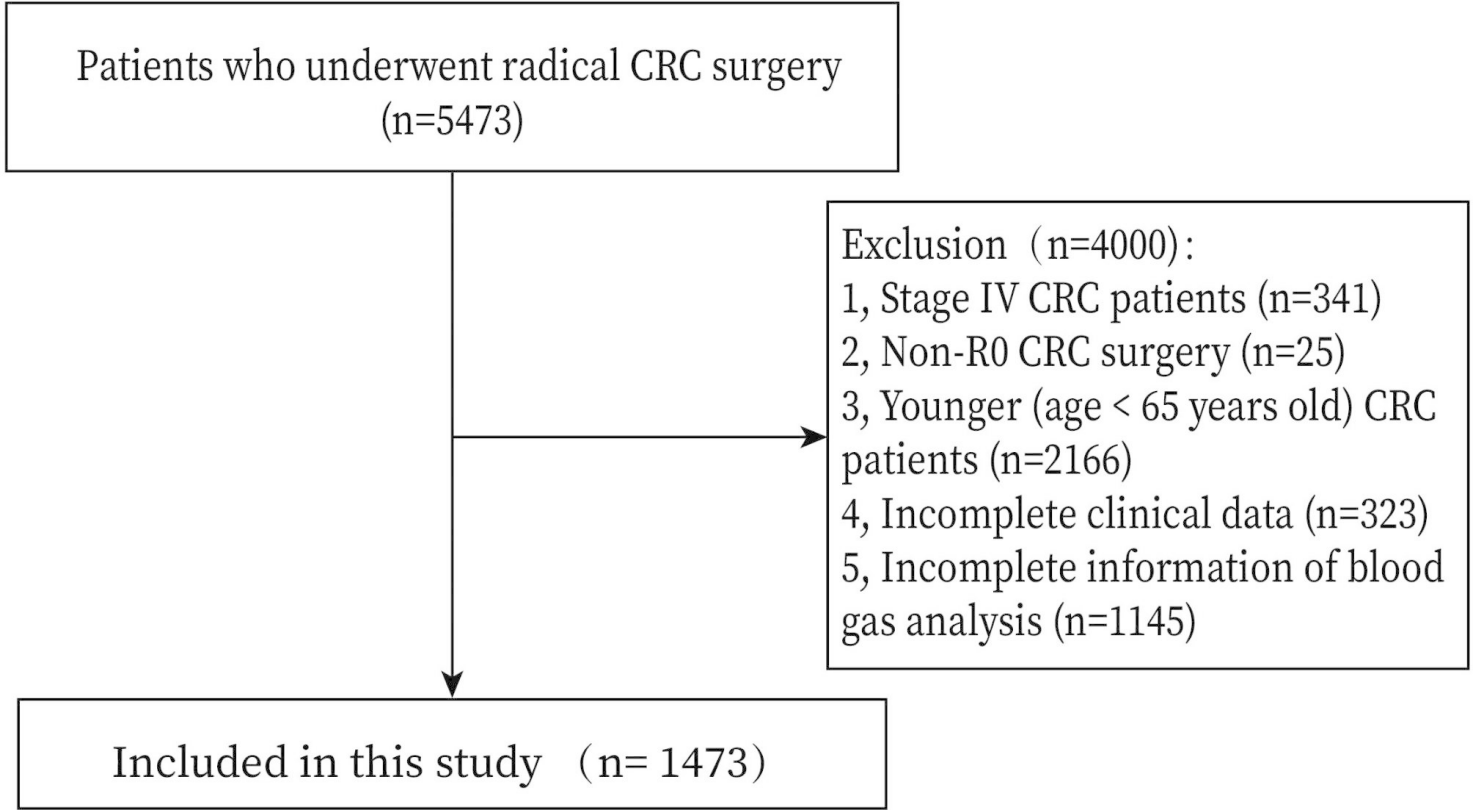
Flow chart of patient selection

We included the patients who underwent radical CRC surgery (n = 5473). The exclusion criteria were as follows: 1, Stage IV CRC patients (n = 341); 2, Non-R0 CRC surgery (n = 25); 3, Younger (age < 65 years old) CRC patients (n = 2166); 4, Incomplete clinical data (n = 323); and 5, Incomplete information of blood gas analysis (n = 1145). Finally, a total of 1473 CRC patients were included in this study. (Fig. 1)

### Clinical data

Clinical data mainly included baseline information and surgery-related data. Baseline information included age, sex, body mass index (BMI), smoking history, drinking history, concomitant disease, tumor location, tumor stage and tumor size. The concomitant diseases mainly included hypertension, type 2 diabetes mellitus (T2DM) and coronary heart disease (CHD). Surgery-related information included surgical method, operative time, blood loss, postoperative hospital stays, retrieved lymph nodes, and postoperative complications. Clinical data were mainly collected through electronic medical record systems.

### Follow-up data

The mean follow-up time was 33 (1-114) months. We routinely followed up by telephone for the first time within 1 month after surgery, then every 3 months for 3 years, and every 6 months thereafter. Follow-up data were obtained primarily through telephone interviews and the outpatient care system.

### Definitions

This study used the X-tile software (version 3.6.1) to determine the optimal cut-off values for bicarbonate and LL [18]. The best cut-off value for bicarbonate was 25.7mmol/L, and the best cut-off value for lactic acid was 0.9mmol/L. Therefore, we defined bicarbonate ≤ 25.7 as the lower group, and > 25.7 as the higher group; lactic acid ≤ 0.9 as the lower group, and > 0.9 as the higher group. Tumor staging was performed according to the TNM in AJCC 8th Edition [19]. The severity of postoperative complications (POCs) was defined according to the Clavien-Dindo classification [20, 21], where Clavien-Dindo ≥ III was defined as major complications. Overall survival (OS) was defined as the time from surgery to the all-cause death or last follow-up in an individual patient, and disease-free survival (DFS) was defined as the time from surgery to radiographic or pathological confirmation of recurrence, death, or the date of the last follow-up.

### Statistical analysis

Continuous variables were expressed as mean ± SD, and frequency variables were expressed as n (%). The above clinical variables were analyzed using independent samples t-test, Fisher’s exact test and Chi-square test by SPSS software (version 22.0). Univariate logistic regression analysis was also performed to find potential predictors of complications, COX regression analysis was performed to identify their independent predictors of OS and DFS. Two-sided P-values less than 0.05 were considered statistically significant.

## Results

### Patient

A total of 1473 patients were included in this study through the inclusion and exclusion criteria. We divided patients into the higher bicarbonate group (916 patients) and the lower bicarbonate group (557 patients) according to the best cut-off value for bicarbonate of 25.7. According to the best cut-off value for LL of 0.9, we divided patients into the higher lactate group (1315 patients) and the lower lactate group (158 patients). We systematically collected the baseline information, surgery-related information and related information of all patients, as shown in Tables 1 and 2.

**Table 1:** Comparison between higher bicarbonate and lower bicarbonate

Characteristics	Higher bicarbonate (916)	Lower bicarbonate (557)	P value
Age, year	72.7 ± 5.8	74.0 ± 6.4	< 0.01*
Sex			0.111
Male Female	563 (61.5%) 353 (38.5%)	319 (57.3%) 238 (42.7%)	
BMI, kg/m^2^	22.4 ± 3.2	22.7 ± 3.4	0.076
Smoking	329 (35.9%)	209 (37.5%)	0.535
Drinking	268 (29.3%)	167 (30.0%)	0.768
Hypertension	334 (36.5%)	220 (39.5%)	0.244
T2DM	160 (17.5%)	99 (17.8%)	0.881
CHD	69 (7.5%)	61 (11.0%)	0.025*
Open surgery	75 (8.2%)	104 (18.7%)	< 0.01*
Tumor location			< 0.01*
Colon	411 (44.9%)	343 (61.6%)	
Rectum	505 (55.1%)	214 (38.4%)	
TNM stage			0.508
I	158 (17.2%)	88 (15.8%)	
II	397 (43.3%)	231 (41.5%)	
III	319 (34.8%)	205 (36.8%)	
IV	42 (4.7%)	33 (5.9%)	
Tumor size			< 0.01*
< 5 cm	552 (60.3%)	282 (50.6%)	
≥ 5 cm	364 (39.7%)	275 (49.4%)	
Operation time (min)	216.3 ± 80.0	221.2 ± 85.9	0.260
Blood loss (mL)	84.3 ± 123.6	103.0 ± 140.5	< 0.01*
Hospital stay (day)	10.8 ± 9.4	11.4 ± 7.7	0.184
Retrieved lymph nodes	15.5 ± 8.0	15.1 ± 6.4	0.260
Overall complications	206 (22.5%)	160 (28.7%)	< 0.01*
Major complications	23 (2.5%)	22 (3.9%)	0.120
30-day deaths	0 (0.0%)	7 (1.3%)	< 0.01*

Note: Variables are expressed as the mean ± SD, n (%), *P-value < 0.05

Abbreviations: T2DM, type 2 diabetes mellitus; BMI, body mass index; CHD, coronary heart disease

### Comparison between the higher group and the lower group

In the baseline information, lower bicarbonate patients were older (p < 0.01), had higher rates of CHD (p = 0.025), a higher proportion of colon tumors (p < 0.01) and larger tumor size (p < 0.01); In the surgery-related data, lower bicarbonate patients had higher rates of open surgery (p < 0.01), more intraoperative blood loss (p < 0.01), higher overall complications (p < 0.01) and 30-day deaths (p < 0.01). We found that there was no statistically significant difference in major complications between the high bicarbonate and low bicarbonate group (P = 0.120). (Table 1)

We found that higher lactate patients had more male patients (p < 0.01), higher BMI (p < 0.01) and drinking rates (p = 0.049), higher rates of T2DM (p < 0.01) and lower rates of open surgery (p < 0.01). We found that there was no statistically significant difference in major complications between the high lactate and low lactate group (P = 0.137). (Table 2)

**Table 2:** Comparison between higher lactate and lower lactate

Characteristics	Higher lactate (1315)	Lower lactate (158)	P value
Age, year	73.2 ± 6.1	73.0 ± 6.0	0.575
Sex			< 0.01*
Male Female	806 (61.3%) 509 (38.7%)	76 (48.1%) 82 (51.9%)	
BMI, kg/m^2^	22.6 ± 3.3	21.5 ± 3.2	< 0.01*
Smoking	487 (37.0%)	51 (32.3%)	0.241
Drinking	399 (30.3%)	36 (22.8%)	0.049*
Hypertension	495 (37.6%)	59 (37.3%)	0.941
T2DM	243 (18.5%)	16 (10.1%)	< 0.01*
CHD	119 (9.0%)	11 (7.0%)	0.382
Open surgery	147 (11.2%)	32 (20.3%)	< 0.01*
Tumor location			0.302
Colon	667 (50.7%)	87 (55.1%)	
Rectum	648 (49.3%)	71 (44.9%)	
TNM stage			0.807
I	222 (16.9%)	24 (15.2%)	
II	555 (42.2%)	73 (46.2%)	
III	471 (35.8%)	53 (33.5%)	
IV	67 (5.1%)	8 (5.1%)	
Tumor size			0.346
< 5 cm	739 (56.2%)	95 (60.1%)	
≥ 5 cm	576 (43.8%)	63 (39.9%)	
Operation time (min)	218.0 ± 78.7	219.7 ± 108.0	0.798
Blood loss (mL)	90.7 ± 130.4	96.5 ± 131.5	0.601
Hospital stay (day)	10.9 ± 8.2	12.3 ± 12.5	0.054
Retrieved lymph nodes	15.4 ± 7.6	15.4 ± 6.7	0.970
Overall complications	317 (24.1%)	49 (31.0%)	0.058
Major complications	37 (2.8%)	8 (5.1%)	0.137
30-day deaths	7 (0.5%)	0 (0.0%)	1.000

Note: Variables are expressed as the mean ± SD, n (%), *P-value < 0.05

Abbreviations: T2DM, type 2 diabetes mellitus; BMI, body mass index; CHD, coronary heart disease

### Univariate and multivariate analysis

We performed multivariate logistic regression analyses and COX regression to identify their independent predictors for complications, OS, DFS. Through analysis, we found that bicarbonate was an influencing factor for overall complications, OS, and DFS, but not an independent risk factor.

Using multivariate logistic regression analysis of overall complications, we found that age (p < 0.01, OR = 1.027, 95% CI = 1.007–1.047), BMI (p = 0.036, OR = 0.961, 95% CI = 0.925–0.997), T2DM (p = 0.023, OR = 1.429, 95% CI = 1.050–1.944), and surgical methods (p < 0.01, OR = 2.124, 95% CI = 1.518–2.970) were independent risk factors. (Table 3)

**Table 3:** Univariate and multivariate logistic regression analysis of the overall complications

Risk factors	Univariate analysis		Multivariate analysis		
	OR (95% CI)	P value	OR (95% CI)	P value	
Age, year	1.035 (1.016–1.055)	< 0.01*	1.027 (1.007–1.047)	< 0.01*	
Sex (male/female)	0.972 (0.764–1.238)	0.820			
BMI, Kg/m^2^	0.957 (0.922–0.992)	0.018*	0.961 (0.925–0.997)	0.036*	
Hypertension (yes/no)	1.086 (0.852–1.384)	0.506			
T2DM (yes/no)	1.450 (1.080–1.948)	0.014*	1.429 (1.050–1.944)	0.023*	
Tumor location (colon/ rectum)	0.981 (0.774–1.242)	0.871			
Tumor stage (IV/III/II/I)	1.183 (1.020–1.372)	0.026*	1.110 (0.953–1.294)	0.179	
Smoking (yes/no)	1.053 (0.825–1.345)	0.677			
Drinking (yes/no)	0.914 (0.704–1.188)	0.502			
CHD (yes/no)	1.086 (0.852–1.384)	0.506			
Tumor size (≥ 5/ <5), cm	1.251 (0.987–1.586)	0.064			
Surgical methods (open/laparoscopic)	2.486 (1.797–3.438)	< 0.01*	2.124 (1.518–2.970)	< 0.01*	
Bicarbonate (higher/lower)	0.720 (0.566–0.915)	< 0.01*	0.805 (0.627–1.033)	0.089	
Lactate (higher/lower)	0.707 (0.493–1.013)	0.059			

Note: *P-value < 0.05

Abbreviations: OR, Odds ratio; CI, confidence interval; BMI, body mass index; T2DM, type 2 diabetes mellitus; CHD, coronary heart disease

Independent risk factors for OS included age (p < 0.01, HR = 1.033, 95% CI = 1.013–1.054), tumor site (p = 0.014, HR = 1.403, 95% CI = 1.069–1.840), tumor stage (p < 0.01, HR = 2.202, 95% CI = 1.848–2.625), tumor size (p = 0.036, HR = 1.327, 95% CI = 1.018–1.730), LL (p < 0.01, HR = 1.981, 95% CI = 1.230–3.191), and overall complications (p < 0.01, HR = 1.656, 95% CI = 1.266–2.167). (Table 4)

**Table 4:** Univariate and multivariate analysis of overall survival

Risk factors	Univariate analysis		Multivariate analysis		
	HR (95% CI)	P value	HR (95% CI)	P value	
Age (years)	1.042 (1.021–1.063)	< 0.01*	1.033 (1.013–1.054)	< 0.01*	
Sex (female/male)	0.861 (0.730–1.015)	0.074			
BMI (kg/m^2^)	0.974 (0.936–1.013)	0.192			
T2DM (yes/no)	1.090 (0.775–1.534)	0.619			
Tumor site (colon/ rectum)	1.510 (1.162–1.963)	< 0.01*	1.403 (1.069–1.840)	0.014*	
Tumor stage (IV/III/II/I)	2.250 (1.896–2.671)	< 0.01*	2.202 (1.848–2.625)	< 0.01*	
Smoking (yes/no)	1.061 (0.813–1.385)	0.662			
Drinking (yes/no)	0.971 (0.728–1.295)	0.840			
Hypertension (yes/no)	0.837 (0.637–1.099)	0.201			
CHD (yes/no)	0.969 (0.939–1.957)	0.890			
Tumor size (≥ 5 cm/<5 cm)	1.690 (1.303–2.192)	< 0.01*	1.327 (1.018–1.730)	0.036*	
Bicarbonate (higher/lower)	0.636 (0.491–0.823)	< 0.01*	0.769 (0.590–1.002)	0.052	
Lactate (higher/lower)	1.818 (1.136–2.911)	0.013*	1.981 (1.230–3.191)	< 0.01*	
Overall complications (yes/no)	1.804 (1.383–2.352)	< 0.01*	1.656 (1.266–2.167)	< 0.01*	

Note: *P-value < 0.05

Abbreviations: HR, hazard ratio; CI, confidence interval; BMI, body mass index; T2DM, type 2 diabetes mellitus; CHD, coronary heart disease

As for DFS, the independent risk factors included age (p = 0.012, HR = 1.024, 95% CI = 1.005–1.042), tumor site (p = 0.019, HR = 1.341, 95% CI = 1.050–1.712), tumor stage (p < 0.01, HR = 2.094, 95% CI = 1.788–2.452), LL (p < 0.01, HR = 2.020, 95% CI = 1.230–3.191), and overall complications (p < 0.01, HR = 1.484, 95% CI = 1.158–1.902). (Table 5)

**Table 5:** Univariate and multivariate analysis of disease-free survival

Risk factors	Univariate analysis		Multivariate analysis		
	HR (95% CI)	P value	HR (95% CI)	P value	
Age (years)	1.032 (1.013–1.051)	< 0.01*	1.024 (1.005–1.042)	0.012*	
Sex (female/male)	0.876 (0.689–1.113)	0.277			
BMI (kg/m^2^)	0.979 (0.945–1.014)	0.243			
T2DM (yes/no)	0.997 (0.727–1.368)	0.986			
Tumor site (colon/ rectum)	1.402 (1.107–1.775)	< 0.01*	1.341 (1.050–1.712)	0.019*	
Tumor stage (IV/III/II/I)	2.129 (1.825–2.485)	< 0.01*	2.094 (1.788–2.452)	< 0.01*	
Smoking (yes/no)	1.050 (0.825–1.338)	0.691			
Drinking (yes/no)	0.968 (0.746–1.257)	0.809			
Hypertension (yes/no)	0.867 (0.678–1.108)	0.253			
CHD (yes/no)	1.001 (0.669–1.499)	0.995			
Tumor size (≥ 5 cm/<5 cm)	1.506 (1.192–1.903)	< 0.01*	1.206 (0.950–1.531)	0.125	
Bicarbonate (higher/lower)	0.723 (0.572–0.914)	< 0.01*	0.859 (0.676–1.092)	0.215	
Lactate (higher/lower)	1.886 (1.219–2.919)	< 0.01*	2.020 (1.230–3.191)	< 0.01*	
Overall complications (yes/no)	1.584 (1.240–2.024)	< 0.01*	1.484 (1.158–1.902)	< 0.01*	

Note: *P-value < 0.05

Abbreviations: HR, hazard ratio; CI, confidence interval; BMI, body mass index; T2DM, type 2 diabetes mellitus; CHD, coronary heart disease

### Complications between the higher group and the lower group

By comparing the complications between higher bicarbonate and lower bicarbonate, we found that lower lactate patients had more overall complications (p < 0.01) and more 30-day deaths (p < 0.01). (Table S1)

As for the higher lactate group and the lower lactate group, we found that higher lactate patients had more re-operation patients (p = 0.035). (Table S2)

## Discussion

A total of 1473 patients were included in this study. Based on the optimal cutoff values for bicarbonate and LL, we divided patients into higher bicarbonate group (916 patients) and lower bicarbonate group (557 patients), higher lactate group (1315 patients) and lower lactate group (158 patients), respectively. The comparison found that the higher lactate group had more male patients, higher BMI and smoking rate, and higher proportion of preoperative diabetes patients.

LL was a valuable prognostic marker in critically ill patients and their dynamics were strongly associated with mortality in surgical patients. [22–24] Under normal physiological conditions, lactate was produced by mitochondria-deficient muscles, skin, brain, gut, and red blood cells at approximately 1500 mmol per day. The metabolism of lactate was mainly carried out in the liver (about 60%), kidneys (about 30%) and other organs [25]. The normal lactate concentration was 1 ± 0.5 mmol/l [26, 27]. Under pathological conditions, other organs such as cardiac muscle, skeletal muscle, lung, white blood cells and splanchnic circulation would produce a large amount of lactic acid, thereby increasing the lactic acid concentration [28, 29]. Multiple previous studies have confirmed the impact of LL on surgery: Hajjar LA et al. found that higher lactate was an independent risk factor for cardiac surgery outcomes, [30] O’Connor E et al. found that LL was associated with longer intensive care unit (ICU) length of stay, [29] and Li SH et al. found that initial serum lactate levels was significantly associated with postoperative complications and independently predicted in-hospital morbidity after major abdominal surgery [31].

Through logistic regression analysis or COX regression analysis of overall complications, OS and DFS, we found that preoperative LL was an independent risk factor for OS and DFS, while bicarbonate had little effect on the prognosis of CRC patients. Acidic extracellular pH was a characteristic of the tumor microenvironment. Bicarbonate neutralized the acidic environment by producing CO_2_. The reduction of bicarbonate might make the microenvironment acidic. The SLC4 protein family was a bicarbonate transporter protein, and the SLC4A4 was a well-characterized acid-extruders [32]. The acid microenvironment caused by bicarbonate reduction also increased the expression of SLC4A4 in colon cancer cell lines [33]. In addition, the acidic tumor microenvironment promoted the degradation of extracellular matrix, further promoted invasion and metastasis, thereby affecting the prognosis of tumor patients [34].

Under hypoxic conditions, mitochondrial conversion of pyruvate was not possible, and thus, lactate was the end product of anaerobic glycolysis [23]. Elevated lactate was often attributed to two main mechanisms: insufficient oxygen levels (e.g., perfusion defects) and lack of anaerobic glycolysis (e.g., altered clearance, drugs, or malignancy). In other words, elevated serum LL was the product of some combination of overproduction and reduced clearance [35, 36].

High lactate concentrations in tumor biopsies were associated with metastasis and poor clinical outcomes. Tumor evolution was influenced by events involving tumor cells and their living environment, termed the tumor microenvironment (TME) [37, 38]. Cancer cells produce excess lactate through anaerobic glycolysis, even in the presence of an adequate oxygen supply, and large amounts of lactate trigger acidification of the TME, leading to immunosuppressive TME, immune escape in the TME, and tumor-associated macrophages formation of cellular (TAM) dysfunction [34, 39–41]. Gu J et al. found that lactate enhanced Treg cell stability and function, while lactate degradation reduced Treg cell induction, increased antitumor immunity, and reduced tumor growth [42]. Therefore, patients in the lower lactate group tend to have a better prognosis.

There was no clear study on whether bicarbonate and LL affected the surgical prognosis of elderly CRC patients, so this study was the first study concerning this topic. However, this study also had certain limitations. First, this study only involved one research center and was a retrospective one; second, the follow-up time of this study was short; finally, the subjects of this study were elderly patients aged ≥ 65 years, with a wide age span. Therefore, a multicenter prospective randomized controlled trial with more detailed groupings should be carried out in the future.

In conclusion, preoperative LL significantly affected postoperative OS and DFS of CRC patients, but bicarbonate might not affect the prognosis of CRC patients. Therefore, surgeons should actively focus on and adjust the LL of patients before surgery.

## Electronic supplementary material

Below is the link to the electronic supplementary material.

Additional File 1: Complications between higher bicarbonate and lower bicarbonate.

Additional File 2: Complications between higher lactate and lower lactate.

## Data Availability

The datasets used and analyzed during the current study are available from the corresponding author on reasonable request.
