# Supplementary material for: Effects of preoperative bicarbonate and lactate levels on short-term outcomes and prognosis in elderly patients with colorectal cancer
Source: BMC Surg. 2023 May 15;23:127. doi: 10.1186/s12893-023-02039-x (PMC10186757; doi:10.1186/s12893-023-02039-x)
Supplement: Supplementary file 1 — Additional File 1: Complications between higher bicarbonate and lower bicarbonate. [file 12893_2023_2039_MOESM1_ESM.docx]

Table S1 Complications between higher bicarbonate and lower bicarbonate

| Characteristics | Higher bicarbonate  (916) | Lower bicarbonate (557) | P value |
| --- | --- | --- | --- |
| Overall complications | 206 (22.5%) | 160 (28.7%) | <0.01* |
| Re-operation | 20 (2.2%) | 10 (1.8%) | 0.609 |
| Bleeding | 5 (0.5%) | 5 (0.9%) | 0.517 |
| Anastomotic leakage | 23 (2.5%) | 14 (2.5%) | 0.998 |
| Pneumonia | 34 (3.7%) | 27 (4.8%) | 0.289 |
| Lymphatic fistula | 10 (1.1%) | 3 (0.5%) | 0.392 |
| Heart problems | 8 (0.9%) | 9 (1.6%) | 0.196 |
| Pulmonary embolism | 3 (0.3%) | 4 (0.7%) | 0.437 |
| Intestinal obstruction | 21 (2.3%) | 22 (3.9%) | 0.067 |
| Venous thrombosis | 6 (0.7%) | 6 (1.1%) | 0.386 |
| Abdominal infection | 32 (3.5%) | 30 (5.4%) | 0.079 |
| 30-day deaths | 0 (0.0%) | 7 (1.3%) | <0.01* |
| Others | 88 (9.6%) | 62 (11.1%) | 0.348 |

Note: Variables are expressed as the mean ± SD, n (%), *P-value <0.05.
