# Supplementary material for: Effects of preoperative bicarbonate and lactate levels on short-term outcomes and prognosis in elderly patients with colorectal cancer
Source: BMC Surg. 2023 May 15;23:127. doi: 10.1186/s12893-023-02039-x (PMC10186757; doi:10.1186/s12893-023-02039-x)
Supplement: Supplementary file 2 — Additional File 2: Complications between higher lactate and lower lactate. [file 12893_2023_2039_MOESM2_ESM.docx]

Table S2 Complications between higher lactate and lower lactate

| Characteristics | Higher lactate (1315) | Lower lactate (158) | P value |
| --- | --- | --- | --- |
| Overall complications | 317 (24.1%) | 49 (31.0%) | 0.058 |
| Re-operation | 23 (1.7%) | 7 (4.4%) | 0.035* |
| Bleeding | 8 (0.6%) | 2 (1.3%) | 0.292 |
| Anastomotic leakage | 30 (2.3%) | 7 (4.4%) | 0.107 |
| Pneumonia | 53 (4.1%) | 8 (5.1%) | 0.538 |
| Lymphatic fistula | 13 (0.9%) | 0 (0.0%) | 0.383 |
| Heart problems | 14 (1.1%) | 3 (1.9%) | 0.416 |
| Pulmonary embolism | 7 (0.5%) | 0 (0.0%) | 1.000 |
| Intestinal obstruction | 39 (3.0%) | 4 (2.5%) | 1.000 |
| Venous thrombosis | 10 (0.8%) | 2 (1.3%) | 0.375 |
| Abdominal infection | 53 (4.1%) | 9 (5.7%) | 0.324 |
| 30-day deaths | 7 (0.5%) | 0 (0.0%) | 1.000 |
| Others | 127 (9.7%) | 23 (14.6%) | 0.054 |

Note: Variables are expressed as the mean ± SD, n (%), *P-value <0.05.
